# Are There Any Developments in the Attitudes and Practices of Oncologists Regarding Fertility Preservation in Saudi Arabia After 12 Years?

**DOI:** 10.7759/cureus.44562

**Published:** 2023-09-02

**Authors:** Mostafa A Arafa, Seham M Abdulkader, Karim H Farhat, Danny M Rabah, Deana K Awartani, Amani A Aldriweesh, Khalid A Awartani, Mashel K Alkahtani, Ghadh A Alsadhan, Doaa A Mohamed

**Affiliations:** 1 Surgery, College of Medicine, King Saud University, Riyadh, SAU; 2 Epidemiology, High Institute of Public Health, Alexandria University, Alexandria, EGY; 3 Obstetrics and Gynaecology, King Faisal Specialist Hospital & Research Centre, King Faisal University, Riyadh, SAU; 4 Urology, King Faisal Specialist Hospital & Research Centre, King Faisal University, Riyadh, SAU; 5 Surgery, King Saud University Medical City, Riyadh, SAU; 6 Family Health, Alexandria University, Alexandria, EGY

**Keywords:** and practices, attitudes, knowledge, cancer in young, saudi arabia, fertility preservation, knowledge attitudes and practices, oncologists

## Abstract

Introduction: The current practice of offering fertility preservation (FP) counseling and treatment has become one of the focal points in patient care throughout cancer treatment. The turning point was the approval of the Council of Senior Religious Scholars four years ago to freeze tissues from the ovarian membrane, the entire ovary, and the eggs for later use in reproduction to preserve the offspring. Thus, we aimed to assess any development in oncologists' knowledge, attitude, and referral practices regarding FP in Saudi Arabia.

Methods: This is a cross-sectional survey using a self-administered questionnaire. We assessed oncologists' opinions on the importance of FP, their perception of the patient's preferences, and factors to consider when discussing the subject. Then, we assessed the knowledge and referral practices, including the timing of referral before starting cancer treatment.

Results: Most oncologists showed good knowledge and positive attitudes toward FP; however, their referral practices could be better. Most were familiar with FP options. The most significant factors influencing the oncologist-patient FP discussion were the number of existing children, marital status, cost, and type of cancer (96.6%, 76.7%, 65.7%, and 58.9%, respectively).

Conclusions: There is a significant improvement in the knowledge and attitude of oncologists toward FP. However, patients' counseling and referral to fertility services still need to be improved. There is a shortfall in the clinical practice guidelines for FP in cancer patients in Saudi Arabia. The implementation of clinical practice guidelines would enhance FP. However, patients' counseling and referral to fertility services still need to be improved. The lack of proper guidelines on FP is affecting oncologists’ practice.

## Introduction

The prevalence of cancer has been increasing among patients worldwide, estimated 19.3 million new cancer cases reported in 2020 by Global Cancer Statistics [1]. Fortunately, current cancer treatment advancements have led to increasing survivorship for patients. However, several cancer treatments are known to damage reproductive organs and cause infertility or sterility, depending on the patient's age and the combination of treatments received [2]. A worldwide incidence of female cancer survivors amounts to 10% of all female survivors under 40 [3,4]. The improvements in survival rates have become a shift of healthcare practice to a more holistic approach to improving cancer survivors' quality of life, as many of the survivors include the reproductive age group; the current practice of offering fertility preservation (FP) counseling and treatment has become one of the focal points in patient care throughout cancer treatment [5]. Considering mental health's importance, it has recently been explored that reproductive and sexual health is among the most common and distressing aspects of cancer survivorship, as discussed in a systemic review trialed in 2022 [6]. The 2011 study by Arafa et al. among oncologists in Saudi Arabia found various knowledge gaps and deficiencies in FP, which affected the oncologists' attitudes and, in turn, were reflected in their subpar practice [7]. The current study aims to assess whether there is any advancement in oncologists' knowledge, attitude, and referral practices toward FP in Saudi Arabia 12 years after the previous study, particularly after the Council of Senior Religious Scientists allowed the freezing of tissues from the ovarian membrane, the entire ovary, and the eggs for later use in reproduction to preserve the offspring, four years ago.

This article was previously posted to the Research Square preprint server on June 13, 2023 (https://doi.org/10.21203/rs.3.rs-2969887/v1).

## Materials and methods

Inclusion criteria: All oncologists with different subspecialties working in hospitals in Saudi Arabia regions.

Exclusion criteria: Other specialties were excluded.

Study design: A cross-sectional study.

Location: The survey was conducted in different regions of Saudi Arabia.

Duration of study: The study was conducted from January to March 2023

Study tools: A self-administered questionnaire and Google Forms were used to collect the required data. All oncologists have been invited to participate in the study. Those who were out of reach were contacted through a Google form. The survey consisted of two parts, the first part assessed the oncologist's opinion of the importance of FP and the perception of patients' preferences regarding FP, in addition to a question about factors that influence the physician-patient discussion of the subject of FP. The second part included questions assessing the knowledge and referral practices, including the timing of referral before starting cancer treatment; different FP options specifically for females, such as oocyte banking, embryo freezing, and ovarian tissue freezing; and the availability of such opportunities in the Kingdom. The same questionnaire, which was employed in 2011, was used in the current study.

IRB details: Approval was granted by the Ethics Committee of the College of Medicine, King Saud University (Date: 15.02.2022 / No: 22/0132/IRB).

Statistical tests: Each percentage answer was calculated using the mean and range of the total number of respondents to each response. Using the chi-square test, categorical variables were compared between groups. To contrast between continuous variables, the student t-test, and the F-test were applied. A significant outcome was defined as having a p-value of less than 0.05.

## Results

A total of 117 oncologists were invited to participate in the study; 14 surveys were excluded due to missing data. Ninety oncologists (87.4%) responded and returned their complete surveys. All respondents were males; their ages ranged from 25 to 55 years. Among the respondents, 62% were medical oncologists, 8.9% were surgical oncologists, and 28.9% were radiation oncologists. Nearly, most of them (84%) were consultants.

Between the three positions of oncologists, there were significant differences in the mean knowledge scale, where radiation oncologists had the higher mean (8.33±1.9), followed by surgical oncologists (7.2±2.1) and medical oncologists (6.8±1.3) (F=3.66 and p>0.001). Meanwhile, no significant difference was detected regarding different age categories (F=1.3 and p<0.55). Neither the oncologists' age nor the number of patients has a significant association with the likelihood of discussion of cryopreservation (X2=1.2 and p=0.76).

The majority of the oncologists (83.3%) perceived the importance of FP as very important. Their level of knowledge and attitude about sperm cryopreservation is good, where 63.3% know where to send patients to cryopreservation, 84.4% know the correct timing of sending patients to do cryopreservation, and 76% of them know about 76% of clinical oncologists respondents know intra-cytoplasmic sperm injection (ICSI). Only 43.3% routinely discuss the process of cryopreservation with their patients in comparison to 50% who rarely or never bring this topic up. The majority of the participants (72%) responded positively when they were asked about the discussion of such topic with the parents of prepubertal patients; however, when asked if they recommend a patient see a reproductive specialist before starting treatment, only 22% mentioned that they refer them to a fertility specialist (Table 1).

**Table 1:** Knowledge, attitudes, and practices of the oncologists toward sperm cryopreservation. ICSI: intracytoplasmic sperm injection

Topic	Response (%)
Oncologists are familiar with ICSI	
Yes	69 (76.66)
No	21 (23.33)
Oncologists know where to send patients for cryopreservation	
Yes	57 (63.33)
No	33 (36.66)
Availability of sperm cryopreservation	
Yes	15 (16.66)
No	75 (83.33)
Timing of sperm cryopreservation	
Before treatment	76 (84.44)
Any time	4 (4.44)
during therapy	6 (6.66)
Not sure	4 (4.44)
Oncologists discuss cryopreservation with the patient	
Routinely	39 (43.33)
Rarely	34 (37.77)
Never	11 (12.22)
If patient asks	6 (6.66)
Perceived importance of cryopreservation	
Very important	75 (83.33)
Just important	14 (15.55)
Not important	1 (1.11)
Referral of the patient to a specialist	
Refer	20 (22.22)
Do not refer	26 (28.88)
Sometimes	44 (48.88)
Discussion of cryopreservation with parents of prepubertal patients	
Yes	65 (72.22)
No	25 (27.77)
Psychological help to patients	
Yes	86 (95.55)
No	4 (4.44)

The number of existing children (96.6%), marital status (76.6%), cost (65.3%), and type of cancer (58.8%) were the most significant factors that influenced physicians to discuss FP with their patients. Religion was the least essential topic that impacted physicians' discussion (Table 2).

**Table 2:** Factors influencing physicians’ discussion of cryopreservation with their patients. Responses are not mutually exclusive.

Factor	Responses (%)
Type of cancer	53 (58.88)
Age of the patient	32 (35.55)
Marital status	69 (76.66)
Number of existing children	87 (96.66)
Cost of sperm cryopreservation	59 (65.66)
Religion	41 (45.55)

Table 3 illustrates the oncologists' attitudes and practices toward female FP. All the respondents were familiar with of female FP option; the most common technique they were familiar with was oocyte cryopreservation (76.6%), followed by ovarian suppression (34.33%). Most oncologists (83.3%) perceived FP as very important, and 92% believed the patients would benefit from referral to a fertility specialist. However, only 25.6% reported that they routinely discuss future fertility with their patients, and nearly the same percentage (27.7%) refer their patients to a fertility specialist. The principal causes for the non-discussion of cryopreservation were the fear of delaying the beginning of the course of therapy (58%) and concern for the future children of cancer patients (31%), as most believed that FP is a complicated process. Only more than have of the respondents (58%) know that oocyte cryopreservation is available in their health facility.

**Table 3:** Knowledge, attitudes, and practices of the oncologists toward female fertility preservation. Responses are not mutually exclusive.

Topic	Response (%)
Which female fertility preservation options are you familiar with?	
Oocyte cryopreservation	69 (76.66)
Ovarian tissue cryopreservation	27 (30)
Ovarian transposition	24 (26.66)
Ovarian suppression	31 (34.33)
Embryonic cryopreservation	28 (31.11)
Perceived importance of fertility preservation	
Very important	75 (83.33)
Just important	14 (15.55)
Not important	1 (1.11)
How often do you discuss future fertility with your patients?	
Always	23 (25.55)
Sometimes	42 (46.66)
Rarely	13 (14.44)
If patient asks	12 (13.33)
Reasons for non-discussion of fertility preservation options with patients	
Emergent needs to start therapy	52 (57.77)
Concern about the well-being of future children	28 (31.11)
Others	10 (11.11)
Is fertility preservation complicated?	
Yes	63 (70)
No	27 (30)
Would the patients benefit from a referral to an infertility specialist for counseling?	
Yes	83 (92.22)
No	7 (7.77)
Do you refer your cancer patients to a specialist?	
Yes	25 (27.77)
No	65 (72.22)
Availability of fertility preservation in physicians’ institute	
Oocyte cryopreservation	53 (58.88)
Ovarian cryopreservation	12 (13.33)
Ovarian transposition	16 (17.77)
Ovarian suppression	34 (37.77)
Embryonic cryopreservation	11 (12.22)

## Discussion

Globally, advancing medical, surgical, and radiation treatment regimens for cancer therapy have improved survival rates, with further positive expectations in the subsequent years. As cancer treatment in the reproductive age group partakes in multiple medical and surgical treatments, cancer survivors often have diminished or absent reproductive capacities concerning the duration and cumulative dosing of treatment regimens [8].

The recent emphasis on FP for reproductive-age cancer patients undergoing treatment is to consider the physiological and emotional well-being and the ability of cancer survivors to reproduce offspring believed to enhance their quality of living after treatment [2].

Most of the oncologists in the current study have a positive attitude toward FP and were also knowledgeable about male and female FP and the correct timing of sending patients to do cryopreservation; most of them were familiar with oocyte cryopreservation, ovarian suppression, and embryonic cryopreservation and had a good perception regarding such topic. However, less than 50% of them discuss the subject with their male patients, while only 25% discuss it with their female patients. This was reflected in the lower percentage of patient referrals to FP (22%-27%).

One of the patient's rights is to discuss and receive complete FP information. Giving cancer patients enough information on preserving their fertility is possible in various ways.

According to a recent study conducted in Oman in 2023, oncologists' understanding of sperm cryopreservation (62%) and pretreatment with gonadotrophin-releasing hormone (GnRH) agonists (47%), in particular, was high. On the other hand, only a small portion of participants (35.3%) and 27%, respectively, were aware of ovarian tissue and oocyte cryopreservation. However, most need more FP information, particularly oocyte and ovarian cryopreservation. In the same context, oncologists in Oman asserted that sending patients to a reproductive specialist would significantly improve the patient's understanding of their fertility problems. They reported that they found, most frequently, patients had utilized sperm cryopreservation (34%) and pretreatment with GnRH agonist (38%) when questioned about how often they encountered cancer patients who had undergone various procedures for FP. However, the majority of medical professionals stated that they had never seen a cancer patient who had employed ovarian tissue cryopreservation (66%), testicular tissue cryopreservation (65%), in vitro fertilization with embryo cryopreservation (59%), or oocyte cryopreservation (59%) [9].

In the study of oncofertility care and influencing factors among cancer patients in Saudi Arabia in 2022, patients have adequate knowledge of FP, yet oncologists occasionally referred such patients to a specific fertility facility, where only 17% have visited a fertility specialist and only 37.8% have received fertility counseling [5]. In the same context, less than half (47%) of oncologists in a USA study referred their patients to an infertility specialist [10], and only 1% of patients were directed to a center for assisted reproduction in a UK study [11].

On the other hand, in a developing country (Mexico), 58% of the physicians always informed their patients about the jeopardy of infertility with cancer therapy, 38% continually discussed FP measures, and 52% always referred patients to fertility specialists [12]. The previous study conducted in Saudi Arabia in 2011 revealed poor knowledge about FP among oncologists, which in turn influenced their practice, where 41 % of the physicians discuss FP with their patients and less than 20% of them refer their patients to a specialist [7].

In comparison with the 2011 study, the possibility of preserving female fertility was unknown to 45% of oncologists; compared to the current study, where all physicians were familiar with many options for female FP, the most common one (77%) was oocyte cryopreservation, and the difference was significant (p<0.05). When questioned about referring patients to a reproductive specialist before starting therapy, nearly half the respondents in the previous study did not refer; compared to the current study, 28.9% did not refer (p<0.05). The percentage of the oncologists in both studies who did not know the sites for FP for men was nearly the same in both studies (32% vs 36.6%), which presents a significant deficit in their knowledge and information and is reflected in their practices, as only a minor portion of young cancer patients have FP facilities. Among the reasons for the low percentage of referrals to fertility specialists was the oncologists' belief that the FP process is complicated and that their patients should start their treatment immediately without delay because of their critical status [7].

According to an Irish national study's findings, 87% of oncologists in the UK said they needed more information regarding FP alternatives. Most said they talked to patients about how treatments might affect their ability to conceive, but only 38% said they often gave them written information. A third one said they did not ordinarily direct patients who had inquiries about fertility to a professional fertility service. Twenty-three percent had never read any FP guidelines. Lack of time, ignorance, perceived low success rates of FP alternatives, poor patient prognosis, and, to a lesser extent, whether the patient already had children, was single, or could not afford FP therapy were the main barriers to starting discussions about FP [13].

The most significant factors influencing the oncologist-patient discussion found in our study were the number of existing children (96.6%), marital status (76.7%), cost (65.7%), and type of cancer (58.9%). Having a patient who has a kid or children and is not concerned about future fertility is impacting their decision to start a discussion of FP with their patients, according to a study done in Oman in 2023, which indicated that more than half of the participants made this statement [9].

In agreement with the current study, the oncologists from the USA stated that the emergent need to start therapy was the leading cause of not discussing FP with their patients [14]. Notably, the same factors that influence the physician-patient discussion of FP in the current study were stated in a 2011 Saudi Arabia survey [7].

Reproduction is a vital topic between medical science and cultural values. Considering local and broader cultural contextual difficulties, it is crucial to pay more attention to how reproductive concerns are handled within the medical oncological practice, along with recommendations for young adult patients and family members of cancer patients involved in fertility and oncological care. Public health regulations that make preservation techniques available to everyone could address the limited access to FP.

## Conclusions

FP is a new topic of high importance in our region, particularly in Saudi Arabia, which could impact family life. After 12 years after the first survey in 2011, it was found that there is a significant improvement in the attitudes and knowledge of oncologists toward FP for cancer patients, with particular gaps in some areas. However, patients' counseling and referral to fertility services are still deficient. There is a shortfall in the clinical practice guidelines for FP in adolescent and young adult cancer patients in Saudi Arabia. Such policies would benefit oncologists and reproductive specialists to upsurge their knowledge of FP for cancer patients and move onward to FP facilities. When counseling, various factors need to be considered, such as pubertal status, partner status, and the need to treat an underlying pathology quickly.

## References

[1] Sung H, Ferlay J, Siegel RL, Laversanne M, Soerjomataram I, Jemal A, Bray F (2021). Global cancer statistics 2020: GLOBOCAN estimates of incidence and mortality worldwide for 36 cancers in 185 countries. CA Cancer J Clin.

[2] Duffy C, Allen S (2009). Medical and psychosocial aspects of fertility after cancer. Cancer J.

[3] McLaren JF, Bates GW (2012). Fertility preservation in women of reproductive age with cancer. Am J Obstet Gynecol.

[4] Dursun P, Doğan NU, Ayhan A (2014). Oncofertility for gynecologic and non-gynecologic cancers: fertility sparing in young women of reproductive age. Crit Rev Oncol Hematol.

[5] Abusanad A, Mokhtar AM, Aljehani SA (2022). Oncofertility care and influencing factors among cancer patients of reproductive age from Saudi Arabia. Front Reprod Health.

[6] Gorman JR, Arthur EK (2022). Chapter 7: Reproductive and sexual health concerns for cancer survivors. Psychological and Medical Perspectives on Fertility Care and Sexual Health.

[7] Arafa MA, Rabah DM (2011). Attitudes and practices of oncologists toward fertility preservation. J Pediatr Hematol Oncol.

[8] Klein CE (2003). Effects of chemotherapy on gonadal function. Holland-Frei Cancer Medicine. 6th edition.

[9] Al Ghaithi A, Al Rashdi E, Al Shukri M, Al Ghabshi R, Albalushi H (2023). Oncologists’ knowledge, practice and attitude toward fertility preservation: a national survey. Life (Basel).

[10] Quinn GP, Vadaparampil ST, Lee JH (2009). Physician referral for fertility preservation in oncology patients: a national study of practice behaviors. J Clin Oncol.

[11] Anderson RA, Weddell A, Spoudeas HA, Douglas C, Shalet SM, Levitt G, Wallace WH (2008). Do doctors discuss fertility issues before they treat young patients with cancer?. Hum Reprod.

[12] Villarreal-Garza C, Martinez-Cannon BA, Barragan-Carrillo R (2021). Physicians’ attitudes, knowledge, and perceived barriers toward fertility preservation in young breast cancer patients in a developing country. Rev Invest Clin.

[13] Adams E, Hill E, Watson E (2013). Fertility preservation in cancer survivors: a national survey of oncologists' current knowledge, practice and attitudes. Br J Cancer.

[14] Forman EJ, Anders CK, Behera MA (2009). Pilot survey of oncologists regarding treatment-related infertility and fertility preservation in female cancer patients. J Reprod Med.

